# *Curcuma amarissima* Extract Activates Growth and Survival Signal Transduction Networks to Stimulate Proliferation of Human Keratinocyte

**DOI:** 10.3390/biology10040289

**Published:** 2021-04-01

**Authors:** Wutigri Nimlamool, Saranyapin Potikanond, Jirapak Ruttanapattanakul, Nitwara Wikan, Siriporn Okonogi, Salinee Jantrapirom, Pornsiri Pitchakarn, Jirarat Karinchai

**Affiliations:** 1 Department of Pharmacology, Faculty of Medicine, Chiang Mai University, Chiang Mai 50200, Thailand; saranyapin.p@cmu.ac.th (S.P.); jirapak.ken@gmail.com (J.R.); salinee.jan@cmu.ac.th (S.J.); 2 Research Center of Pharmaceutical Nanotechnology, Faculty of Pharmacy, Chiang Mai University, Chiang Mai 50200, Thailand; siriporn.okonogi@cmu.ac.th; 3 Institute of Molecular Biosciences, Mahidol University, Salaya, Nakorn Pathom 73170, Thailand; nitwara.wik@mahidol.edu; 4 Department of Pharmaceutical Sciences, Faculty of Pharmacy, Chiang Mai University, Chiang Mai 50200, Thailand; 5 Department of Biochemistry, Faculty of Medicine, Chiang Mai University, Chiang Mai 50200, Thailand; pornsiri.p@cmu.ac.th (P.P.); jirarat.ka@cmu.ac.th (J.K.)

**Keywords:** human epidermal keratinocytes, HaCaT, *Curcuma amarissima*, PI3K/AKT, RAS/ERK, wound healing, proliferation, survival

## Abstract

**Simple Summary:**

Like many plants in the family of Zingiberaceae, *Curcuma amarissima* has been traditionally used to induce healing and tissue regeneration. However, there is no scientific evidence to explain how *Curcuma amarissima* works to accelerate wound healing. Our data clearly proved that *Curcuma amarissima* extract could potentially accelerate the closure of scratch wounds of human keratinocytes by stimulating cell proliferation. The potential mechanisms underlying these effects were defined to be associated with the activated signal transduction pathways relevant to cell proliferation and survival. This strongly suggests the ability of *Curcuma amarissima* to enhance the process of keratinocyte reepithelization during wound healing. Our current study provides convincing evidence that supports the possibility to develop an effective wound-healing promoting agent from this plant.

**Abstract:**

Many medicinal plants have been used to treat wounds. Here, we revealed the potential wound healing effects of *Curcuma amarissima* (CA). Our cell viability assay showed that CA extract increased the viability of HaCaT cells that were cultured in the absence of serum. This increase in cell viability was proved to be associated with the pharmacological activities of CA extract in inducing cell proliferation. To further define possible molecular mechanisms of action, we performed Western blot analysis and immunofluorescence study, and our data demonstrated that CA extract rapidly induced ERK1/2 and Akt activation. Consistently, CA extract accelerated cell migration, resulting in rapid healing of wounded human keratinocyte monolayer. Specifically, the CA-induced increase of cell monolayer wound healing was blocked by the MEK inhibitor (U0126) or the PI3K inhibitor (LY294002). Moreover, CA extract induced the expression of Mcl-1, which is an anti-apoptotic protein, supporting that CA extract enhances human keratinocyte survival. Taken together, our study provided convincing evidence that *Curcuma amarissima* can promote proliferation and survival of human keratinocyte through stimulating the MAPK and PI3K/Akt signaling cascades. These promising data emphasize the possibility to develop this plant as a wound healing agent for the potential application in regenerative medicine.

## 1. Introduction

Ineffective skin wound healing is becoming a big problem in the public health sector. Several factors including aging, diabetes, infection, immunodeficiency, and cancers can lead to unsuccessful wound treatment and eventually cause morbidity and mortality [1,2,3,4,5]. Although the human body has a great protective skin barrier, sometimes an unexpected injury is unavoidable. A process of organisms, namely “wound healing”, is responsible for the reconstruction of injured skin. This physiological repairing system requires four stages, which include hemostasis (blood clotting), inflammation, proliferation (growth of new tissue), and maturation (remodeling) [6,7]. A successful step of re-epithelialization is considered to be an essential indicator of wound closure to prevent further infection and chronic wound development [8]. In response to skin damage, the process called re-epithelialization is triggered to restore the damaged epidermis. The most important cell types responsible for re-epithelialization are keratinocytes, which proliferate, differentiate, and migrate to heal the open wound [9]. Failure of keratinocytes to maintain skin integrity and/or wound closure could cause reoccurrence of affected lesions and further complicated skin reaction [10]. At the cellular and molecular level, growth and survival signaling cascades are very crucial for enhancing certain stages of re-epithelialization. Specifically, the signaling pathway of mitogen-activated protein kinase (MAPK) is involved in regulating cell proliferation and migration [11,12]. In particular, ERK1/2 kinase phosphorylation is induced by various groups of growth factors [13,14,15,16]. Therefore, certain growth factors such as EGF or PDGF are approved to be used to stimulate the skin healing process [17,18,19]. Nevertheless, growth factors (used at high concentrations) can cause adverse effects, including uncontrolled cell growth, chronic inflammatory skin disease such as psoriasis, or aberrant skin functions [20,21]. Therefore, discovering novel wound healing agents that have lesser side effects would be beneficial for patients with certain conditions.

Since ancient times, people have used herbal medicines as one of the components in wound management to accelerate wound healing. In particular, for treatments derived from plants, both topical and systemic herbal agents have been widely used in wound healing. Several properties, including anti-inflammatory, antioxidant, and antimicrobial activities, are required for being effective wound healing agents [22,23]. Thus, any herbs with these properties will be worth investigating and using for the development of effective wound healing agents. Generally, extracts or the isolated phytochemical compounds derived from plants promote the tissue regeneration process via certain mechanisms, which ultimately provide a synergistic effect on healing efficiency [24]. Currently, some natural products that include herbs [25] have been included as active components for wound treatments. For instance, it has been demonstrated that *Poincianella pluviosa* extract could potently accelerate keratinocyte migration and proliferation [26]. Recently, *Boesenbergia rotunda* or fingerroot, belonging to the Zingiberaceae family (same family with *Curcuma amarissima*) has been revealed to promote human keratinocyte proliferation via stimulating the phosphorylation of ERK1/2 and Akt [27]. Although many plant-derived medicines are believed to be effective, affordable, and cause minimal unwanted side effects [28], it is necessary to carefully define the exact mechanisms of action to be certain how medicinal plants function.

*Curcuma amarissima* Roscoe (CA) or “black turmeric” or “Kamin-dum” is in the family Zingiberaceae and is usually used to treat amoebic dysentery, enteritis, and vermicide [14]. Lectin isolated from the rhizomes of this plant showed anti-fungal activity against several different species, including *Colectrotricum cassiicola, Exserohilum turicicum,* and *Fusarium oxysporum*. Moreover, the purified lectin could also reduce cell proliferation in a breast cancer cell line, BT474 [29]. Accumulating evidence has suggested that plants in the Zingiberaceae family may be one of the great candidates for wound healing [27,30,31]. Specifically, several activities of herbs in this family contain essential combinations that are necessary for the healing process. Those activities include antioxidant, antimicrobial, anti-inflammation, promoting collagen production, promoting dermal cell proliferation, promoting new blood vessels, and central/peripheral antinociceptive effects [32,33]. Previous studies disclosed that the rhizomes of CA contain several different active constituents similar to those found in *Curcuma longa*. Those compounds include curcumenol, curdione, curzerenone, germacene, isofrtungermacrene, and zedoarone [34].

Although many plants in the family of Zingiberaceae have been revealed to have promising effects suitable for wound treatment, there is no evidence showing that *Curcuma amarissima* Roscoe has regenerative effects on skin. Therefore, it is of our interest to investigate whether the *Curcuma amarissima* Roscoe extract has specific pharmacological activities that help enhance wound healing processes.

Here, we discovered that CA can enhance human keratinocyte, HaCaT, cell proliferation and migration via inducing ERK1/2, and Akt phosphorylation, which are important molecular pathways involved in re-epithelialization. Our current study provided information that CA can be developed as an agent for accelerating skin wound repair.

## 2. Materials and Methods

### 2.1. Preparation of Ethanolic Extract from the Rhizomes of Curcuma aeruginosa (CA)

The rhizomes of *Curcuma amarissima* Roscoe were obtained from the cultivating areas in Mae Taeng District, Chiang Mai, Thailand, and were identified by a botanist at the Faculty of Pharmacy, Chiang Mai University. The samples of authenticated *Curcuma amarissima* Roscoe were deposited in the Herbarium of the Faculty of Pharmacy, Chiang Mai University, with the voucher specimen number 0023261. For preparing the ethanolic extract, the fresh rhizomes of *Curcuma amarissima* Roscoe were washed, cut into small pieces, dried, and ground. Next, the ground powder was mixed with ethanol (95%) at room temperature (RT) for 24 h. The mixture was filtered through Whatman No.1 filter paper (Sigma-Aldrich, Saint Louis, MO, USA), and the filtered solution was subjected to a rotary evaporator at 40 °C to eliminate the solvent. Next, one gram (g) of the obtained CA extract was diluted in 1 milliliter (mL) 100% dimethyl sulfoxide (DMSO) and used as a stock solution. For each treatment, the CA extract stock solution (1 g/mL in DMSO) was further pre-diluted in medium to obtain the final working concentrations. However, the final concentration of DMSO was not allowed to exceed 0.5% *v*/*v* in the diluted media throughout the experiment.

### 2.2. HPLC Fingerprint of CA Extract

HP1100 system (HPLC LC-10, Shimadzu, Kyoto, Japan) with an Agilent C-18 column (150 × 4.6 mm, 5 µm) was applied for visualizing the HPLC fingerprint of the extract. The system was performed with a thermostatically controlled column oven and a UV detector set at 254 and 360 nm. The mobile phase was methanol–water system with gradient elution as follows: 40–70% methanol for 0–30 min, 100% methanol for 80–100 min, 40% methanol for 105–115 min. The extract was diluted with methanol to 50 mg/mL before injection (10 µL of sample volume) into the column, with 1.0 mL/min of flow rate.

Additionally, quantitative analysis of curcuminoids and polyphenolic contents in CA extract by HPLC was performed. CA extract was determined for the existence of curcuminoid and polyphenolic contents by HPLC using a C18 column (250 × 4.6 mm, 5 μm) (Agilent Technologies, Santa Clara, CA, USA). The detection of curcuminoids, including bis-demethoxycurcumin, demethoxycurcumin, and curcumin, was carried out using isocratic mode of mobile phase (2% acetic acid in water and acetonitrile 50:50 *v*/*v*) with a flow rate of 1 mL/min. Ten microliters of the extract (20 mg/mL dissolved in 1 mL of MeOH) was injected into the column, and detection was done at 425 nm. The polyphenolic content in the extract was determined by using a gradient system of mobile phase A (1% acetic acid in water) and mobile phase B (100% acetonitrile) with a total run time of 50 min for the detection with a flow rate of 0.7 mL/min and detection wavelength at 280 nm. The gradient system used was 90% A in 0 min–60% in 28 min, followed by 40% in the next 39 min and 10% in the next 50 min.

### 2.3. Culture of Human Keratinocyte Cell Line, HaCaT

HaCaT cell line was purchased from CLS Cell Lines Service GmbH (CLS Cell Lines Service GmbH, Eppelheim, Baden-Wurttemberg, Germany) and maintained in DMEM (Gibco, New York, NY, USA), supplemented with 10% (*v*/*v*) fetal bovine serum (FBS) (Gibco, New York, NY, USA), 100 U/mL penicillin and 100 μg/mL streptomycin) (Gibco, New York, NY, USA) at 37 °C in a humidified atmosphere of 5% CO_2_. For experiments that required serum deprivation, cells were cultured in serum-free media.

### 2.4. Cell Viability Determination of HaCaT Cells Treated with CA Extract

MTT assay was conducted in CA-extract-treated HaCaT cells, which were cultured in either media containing fetal bovine serum (FBS) or FBS-free media. The viability characteristics of the cell were observed. Moreover, toxic and non-toxic concentrations of CA were obtained. Briefly, we cultured (5 × 10^4^ cells/well in 96-well plates) in complete media overnight. For CA extract treatment, the CA extract stock (1 g/mL) was pre-diluted in FBS-free media to make a final concentration of 160 μg/mL (containing DMSO at 0.16%). DMSO (as a vehicle control) was diluted in the same manner. Next, we prepared the treating media containing various concentration of CA extract (ranging from 160 down to 0.625 μg/mL) and DMSO (0.000625–0.16%) by making a two-fold dilution of CA extract or DMSO in either FBS-rich or FBS-free media. Cells were treated with individual treating media (200 μL/well in 96-well plates) for 48 h and subjected to a working solution (0.4 mg/mL) of MTT reagent for 1 h in an incubator. After MTT was discarded, 100 µL of DMSO was added to each well, and the developed color was detected (at 570 nm) by an absorbance reader plate spectrophotometer (BioTek Instruments, Winooski, VT, USA). From this experiment, we chose three concentrations (2.5, 5, and 10 µg/mL) of CA extract for all experiments. Moreover, the proliferative effect of CA extract at specific non-toxic concentrations on HaCaT cells was determined by phase-contrast microscopy. For this experiment, cells were treated with CA extract (2.5, 5, and 10 µg/mL) in FBS-rich media or FBS-free media, and the changes in the size of cell colonies were captured at various time points (0, 24, 48, and 72 h). In some experiments, HaCaT cells were fixed and permeabilized with absolute methanol and stained with crystal violet to clearly verify the density and the size of the colonies.

### 2.5. Determination of HaCaT Cell Monolayer Healing by Scratch Wounding Assay

Wound healing assay of HaCaT cell monolayer was performed. Briefly, confluent HaCaT cells cultured in 24-well plate were scratched by a P200 pipette tip to make a thin wound. Scratch wounds were created in confluent monolayers using a P200 disposable micropipette tip. Cells were then treated with CA extract at all three non-toxic concentrations diluted in serum-deprived media, with or without 10 μM U0126 (CST, Boston, MA, USA) or 50 μM of LY294002 (CST, Boston, MA, USA). The rate of keratinocyte migration was monitored over time, and representative pictures were taken at different time points (0, 20, 30, 40, and 50 h). The measured wound areas were analyzed by the ImageJ program.

### 2.6. Effects of CA Extract on Increasing Number of HaCaT Cells

We directly counted total number of CA extract-treated cells to confirm the effects of CA extract on inducing keratinocyte proliferation. Low density of cells (2.5 × 10^4^ cells/well) was seeded in 24-well plates overnight. Then, media were changed to serum-free media before treating cells with 10 μg/mL. At each incubation time point (0, 24, and 48 h), cells were detached from the plate and counted. The differences in the number of cells between control and experimental groups were calculated.

### 2.7. Effects of CA Extract on Stimulating Molecular Signaling Pathways

To investigate the responsible molecular signaling in which CA extract can be activated, Western blot analysis was conducted to detect certain molecular players responsible for conveying signal transduction for cell proliferation and survival. Specifically, cells were seeded in complete media in 3-cm dishes for 24 h. Cells were then cultured in media without serum for 24 h. For a positive control, HaCaT cells were treated with 100 ng/mL of EGF for 15 min before harvesting. For determining whether CA extract stimulates the early signaling, we treated HaCaT cells with 10 μg/mL of CA extract and harvested them at a certain time (0 min to 24 h). For evaluating the concentration-dependent effects of CA extract, we treated HaCaT cells with 3 different non-toxic concentrations of CA extract for 15 min (with or without U0126 or LY294002). For any experiment involving inhibitors, cells were pre-treated with inhibitors (10 μM of U0126 or 50 μM of LY294002) for 2 h prior to CA extract addition. For the experiment that aimed to detect the level of Mcl-1 protein, cells were treated with CA extract at 2.5, 5, 10 μg/mL, or DMSO as a vehicle control, for 24 h before harvesting cells. The effects of CA on inducing the expression and activating the phosphorylation of ERK1/2 (pERK1/2), and Akt (pAkt), cells were treated with CA extract (10 μg/mL) for 30 min. After preparing cell lysates, all samples were subjected to SDS-PAGE and Western blot analysis. Membranes were then incubated with 5% skim milk dissolved in TBST (Sigma-Aldrich, Saint Louis, MO, USA) at room temperature (RT) for 1 h. After washing trice, membranes were incubated with primary antibodies for 24 h at 4 °C. Primary antibodies (Cell Signaling Technology (CST), Boston, MA, USA) included (1) an anti-pErk1/2 antibody, (2) an anti-total Erk1/2 antibody, (3) an anti-pP38 antibody, (4) an anti-total p38 antibody, (5) an anti-pSAPK/JNK antibody, (6) an anti-total APK/JNK antibody, (7) an anti-pAkt antibody, (8) an anti-total Akt antibody, (9) an anti-pPRAS40 antibody, (10) an anti-pEGFR antibody, (11) an anti-total EGFR antibody, (12) an anti-Mcl-1 antibody, and a an anti-β-actin antibody. Next, membranes were incubated with appropriate secondary antibodies (Li-COR Biosciences, Lincoln, NE, USA), which were an anti-mouse IgG (conjugated with IRDye^®^800CW) or an anti-rabbit IgG (conjugated with IRDye^®^680RT) for 2 h, at RT. The Western blot signal was detected with Odyssey^®^ CLx Imaging System (LI-COR Biosciences, Lincoln, NE, USA), and the intensity of each immunoreactive band was analyzed by Image J.

### 2.8. Detection of Early Signaling Pathway Induced by CA Extract in Individual Cells by Immunofluorescence Study

The phosphorylation of key kinases including ERK1/2 (pERK1/2), Akt (pAkt), EGFR (pEGFR at tyrosine 1068), and the expression of total EGFR and Mcl-1 proteins upon CA extract stimulation were determined in individual cells by immunostaining technique. Sample cover slips were prepared by seeding HaCaT cells on glass cover slips to confluence, and cells were then treated with CA extract (10 µg/mL) for 30 min. Next, fixation was performed, 15 min at RT, using 4% paraformaldehyde (Sigma-Aldrich, Saint Louis, MO, USA). After washing three times with PBS, 0.3% Triton X-100 was added to permeabilize cells for 5 min. Sample cover slips were then blocked with 1% BSA for 1 h at RT and incubated with primary antibodies overnight at 4 °C. Primary antibodies were an anti-pErk1/2 antibody, an anti-pAkt antibody, an anti-pEGFR receptor antibody, an anti-total EGFR antibody, and an anti-Mcl-1 antibody. Sample coverslips were then probed with anti-rabbit IgG (Alexa488-conjugated) or goat anti-rabbit IgG (Alexa594-conjugated) (Thermo Fisher Scientific, Waltham (HQ), MA, USA) for 2 h at RT. The nuclei of HaCaT cells were stained with DAPI (1 μg/mL) (Sigma-Aldrich, Saint Louis, MO, USA). Sample coverslips were washed three times with PBS, and one time with distilled water before being subjected to mounting with Fluoromount-G (SouthernBiotech, Bermingham, AL, USA) as a mounting medium. Positive fluorescent signals were detected and recorded by a fluorescence microscope, Axio Vert.A1 (Carl Zeiss Suzhou Co. Ltd., Suzhou, China) equipped with Colibri 7 illumination system (Carl Zeiss Microscopy GmbH, Gottingen, Germany) and Axiocam 506 color digital camera (Carl Zeiss Microscopy GmbH, Gottingen, Germany) using immersion objective (100×/1.3 Oil M27). Signal of the nuclei stained with DAPI was excited by the UV mode (385/30 nm), signal of Alexa488 was excited by the blue light mode (469/38 nm), and signal of Alexa594 was excited by the green mode (555/30 nm). Representative pictures were taken with the Zen 2.6 (blue edition) Software.

### 2.9. Data and Statistical Analysis

Results were presented as mean ± standard deviation (SD). One-way analysis of variance (ANOVA) was performed, followed by Tukey’s post hoc multiple comparisons (SPSS Inc., Chicago, IL, USA). *p* values less than 0.05 were considered statistically significant.

## 3. Results

### 3.1. Curcuma Amarissima (CA) Extract Enhances Cell Viability of HaCaT Cells

**Figure 1:**
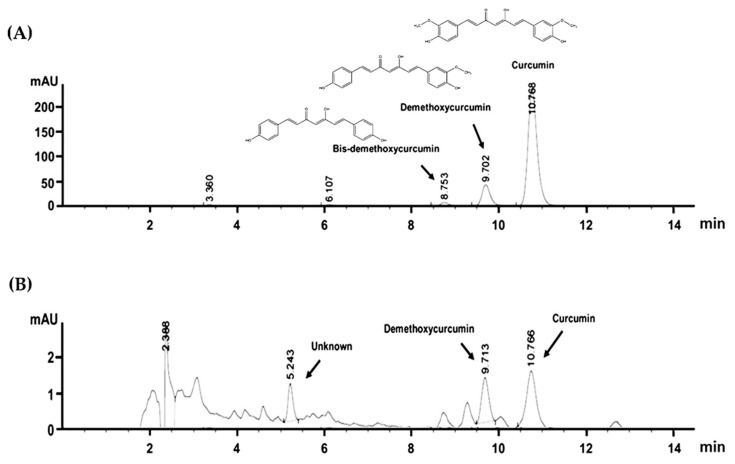
The HPLC profile of (**A**) curcuminoids (50 μg/mL) including bis-demethoxycurcumin, desmethoxycurcumin, and curcumin with their chemical structures. (**B**) The HPLC profile of *Curcuma amarissima* extract (20 mg/mL) indicating the existence of desmethoxycurcumin and curcumin.

After obtaining the extract, we performed chromatographic fingerprint analysis of the ethanolic extract from *Curcuma amarissima* (CA) by high-performance liquid chromatography (HPLC). The results (both detected at 254 and 360 nm) showed the unique characteristics of compound fingerprint in CA extract (Figure S1). Like many plants in the genus *Curcuma* such as *Curcuma longa, Curcuma amarissima* may contain some active compounds including curcumin that has been shown to have significant wound healing properties. Therefore, quantitative analysis of curcuminoids in *Curcuma amarissima* extract was examined by HPLC. As expected, the results revealed the existence of two curcuminoids in the extract which were desmethoxycurcumin (5.87 ± 0.001 µg/g extract) and curcumin (10.81 ± 0.001 µg/g extract) (Figure 1). We also found that the extract contained ferulic acid (Figure S2).

**Figure 2:**
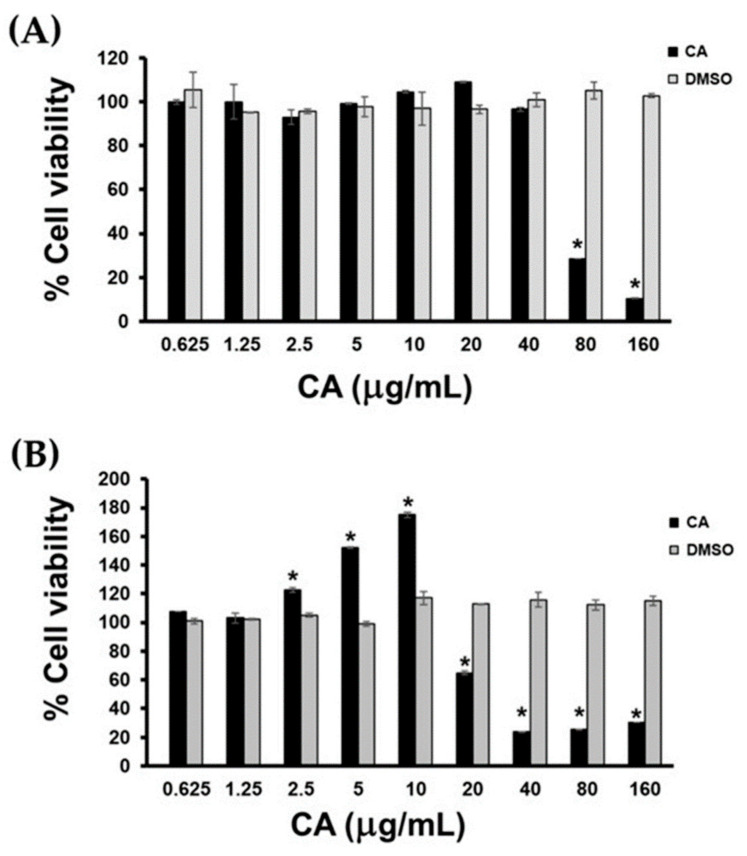
Cell viability HaCaT cells upon treatment with *Curcuma amarissima* (CA) extract. Viability of HaCaT cells treated with CA extract (0.625 to 160 μg/mL) or DMSO (0.000625–0.16%) for 48 h in FBS-rich media (**A**) and in FBS-free media (**B**). Data from three experiments were analyzed and presented as mean ± standard deviation (SD); * *p* < 0.05 in comparison to the untreated group.

We performed MTT cell viability assay to monitor changes in cellular metabolic activity and viability of HaCaT cells in media containing CA extract (CA extract in DMSO as a solvent from the stock solution) with the presence of 10% Fetal Bovine Serum, FBS. Cells were also treated with the FBS-rich media containing DMSO (as a vehicle control) with varied concentrations corresponding to those present in the CA extract-treated group. Results demonstrated that CA extract at concentrations lower than 40 µg/mL had no significant effect on the viability of HaCaT cells cultured in FBS-rich media (Figure 2A). However, the extract at 80 and 160 µg/mL caused significant reduction in cell viability to approximately 30% and 10%, respectively. However, DMSO at all concentrations used did not cause any change in HaCaT cell viability (Figure 2A). We also performed similar experiments where treatment with CA extract was done in FBS-free media. Results showed that HaCaT cell viability in serum-free media was more sensitive to CA treatment. In particular, when cells were treated with CA at 20 µg/mL, cell viability was observed to be significantly reduced, and the cell viability was maximally decreased to approximately 20% in cells treated with CA at 40 µg/mL or more (Figure 2B). Interestingly, we observed that HaCaT cell metabolic activity dramatically increased in response to treatment with CA extract at 2.5, 5, and 10 µg/mL (Figure 2B). The results indicate that CA extract induced HaCaT cell metabolic activity, and this activity may be caused by an increasing number of cells upon CA extract treatment.

### 3.2. CA Extract Stimulates Colony Formation and Proliferation of HaCaT Cells

**Figure 3:**
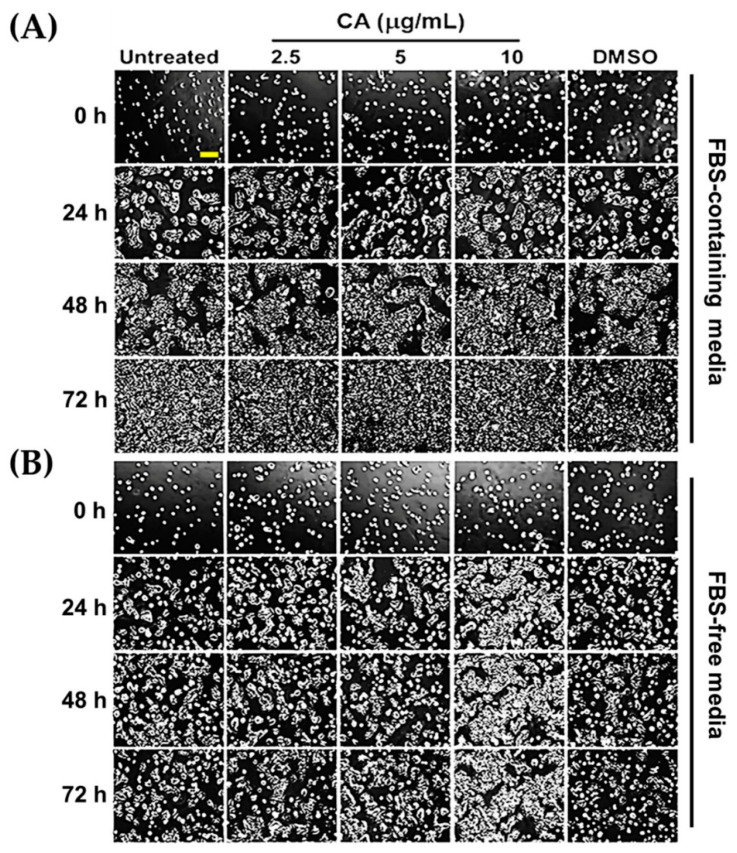
CA extract induced the growth of HaCaT colonies. HaCaT cells were left untreated or treated with CA extract at 2.5, 5, and 10 g/mL in media containing 10% FBS (**A**) or in media without the presence of FBS (**B**). The sizes of HaCaT colonies were monitored, and the pictures were captured at 0, 24, 48, and 72 h by a phase-contrast microscope (10× magnification, scale bar = 200 μm). Cells were also treated with DMSO which served as a vehicle control. The pictures were representative of three individual experiments.

From the previous experiment, data clearly showed that CA extract at non-toxic concentrations (2.5, 5, and 10 µg/mL) could significantly induce HaCaT cell metabolic activity in a concentration-dependent fashion, which could be seen when serum was deprived. We thought that an increase in cell viability of HaCaT cells may have resulted from the proliferative effects of CA extract. Therefore, we observed whether CA extract can accelerate the growth of the HaCaT colonies over time by a phase-contrast microscope. Our data demonstrated that at 0 h of CA treatment or DMSO vehicle control (in both complete and serum-free media), cells were detected to be single cells with equal distribution and similar cell density on the surface of the dish (Figure 3A,B). Over the course of 72 h, we noticed that the colony size of HaCaT cells incubated with CA extract in FBS-rich media gradually increased from single cells at 0 h to 100% confluence at 72 h (Figure 3A). We also observed that the colonies of CA-extract-treated cells (at 10 µg/mL) were slightly larger than those of the untreated or DMSO-treated cells (Figure 3A). These observations were clearly verified by the results obtained from CA treatment in serum-free media, where HaCaT cells were suppressed to undergo slow cell proliferation due to the lack of growth factors in FBS that generally stimulate cell proliferation. Specifically, CA extract at 10 µg/mL showed strong effects on accelerating the growth of the HaCaT colonies at 24, 48, and 72 h of incubation (Figure 3B).

**Figure 4:**
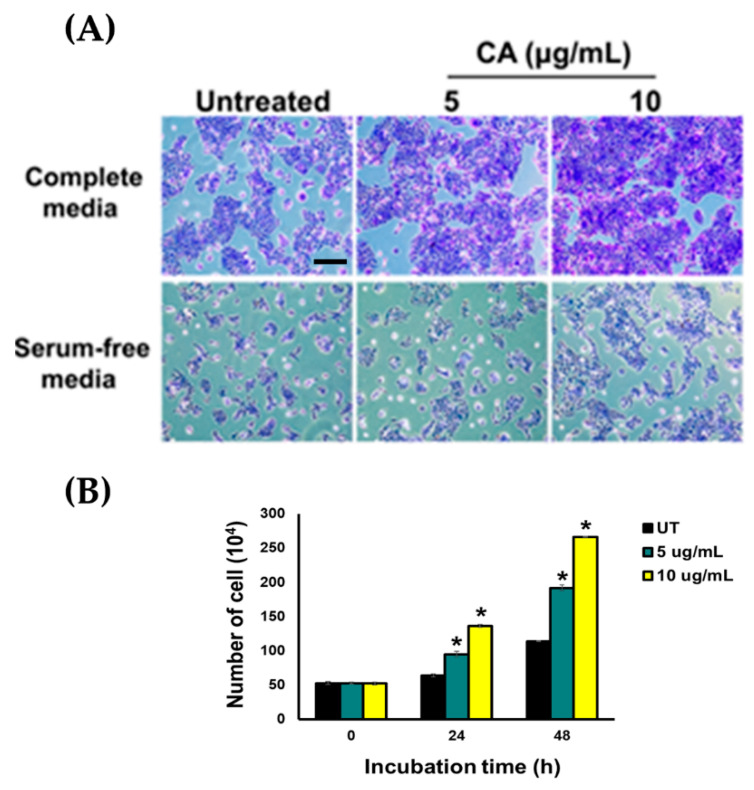
(**A**) Crystal violet staining of CA-extract-treated cells (5 and 10 μg/mL) for 24 h in FBS-rich media or FBS-free media detected by a phase-contrast microscope (10× magnification, scale bar = 200 μm). (**B**) Analysis of total number of cells treated with CA extract at 5 and 10 μg/mL over the course of 24 and 48 h in FBS-free media. Data, from three experiments, present mean ± SD; * *p* < 0.05 (in comparison to the untreated (UT) group).

We attempted to clarify whether the observed phenomenon is an effect of CA extract on stimulating cell proliferation. Therefore, we treated HaCaT cells with CA extract (5 and 10 µg/mL) in media containing FBS or FBS-free media for 2 days, and then cells were stained with crystal violet and we observed clear phenotypic changes of the HaCaT colonies (Figure 4A). Consistent with these results, our cell counting assay revealed that CA extract exhibited the ability to stimulate an increase in cell number over 48 h (Figure 4B).

### 3.3. CA Extract Induces Migration of HaCaT Cell Monolayer into The Wounded Area

**Figure 5:**
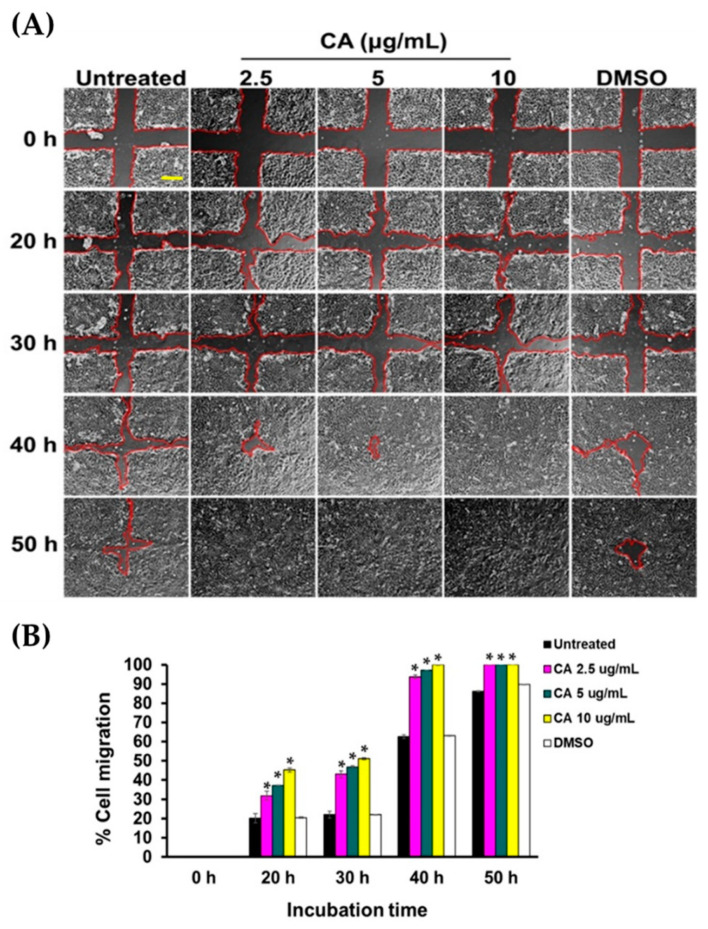
CA extract stimulated HaCaT cell monolayer healing. (**A**) HaCaT cell monolayer treated with CA extract at various concentrations in FBS-free media was monitored for the ability to heal over the course of 50 h by a phase-contrast microscope (10× magnification, scale bar = 200 μm). The vehicle control group was treated with DMSO. (**B**) The analysis of percent migration of CA extract-treated HaCaT cells in FBS-free media at each time point (0, 20, 30, 40, or 50 h). Regions confined by the red lines indicate the space with no cell occupation. Data present mean ± SD (* *p* < 0.05, compared to the untreated).

Besides proliferation, migration of human keratinocytes is an additional crucial step that contributes to efficient healing. To test that CA extract can stimulate HaCaT cells migration, we monitored the rate of migration into the wounded area of keratinocytes treated with CA extract at varied concentrations and time points. Our quantitative analysis clearly demonstrated that CA extract significantly promoted percent cell migration into the wounded areas in a concentration-dependent manner and in all time points (20, 30, 40, and 50 h), compared to those of the untreated and DMSO-treated groups (Figure 5A,B).

### 3.4. Proliferation and Survival Signal Transductions Are Induced in HaCaT Cells upon Treatment with CA Extract

**Figure 6:**
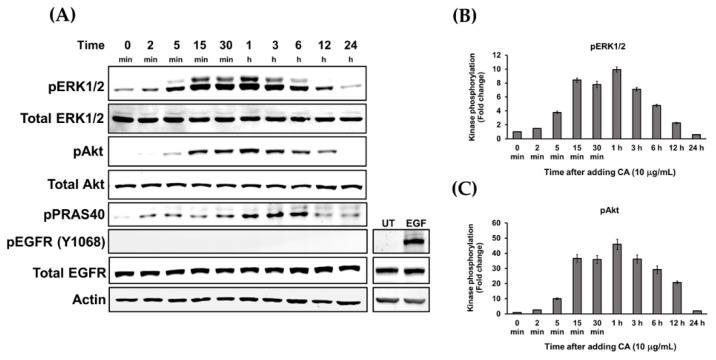
CA extract strongly stimulated ERK1/2 and Akt phosphorylation. (**A**) Time-dependent detection by Western blot analysis for the expression and phosphorylation of crucial signaling molecules (ERK1/2, Akt, PRAS40, and EGFR) in HaCaT cells treated with 10 µg/mL of CA extract over 24 h. (**B**) Quantification of the phosphorylation of ERK1/2 in CA extract-incubated HaCaT cells. (**C**) Quantification of the phosphorylation of Akt in CA extract-treated HaCaT cells. Actin was used as a loading control. Data from three individual experiments present mean ± SD; *p* < 0.05.

Since we observed that CA induced HaCaT cell proliferation and migration, we hypothesized that CA extract stimulates growth and survival signaling in HaCaT cells. Therefore, we tested our hypothesis by focusing on relevant molecular signaling cascades. It is well-defined that the MAPK signaling pathway is important for activating cell proliferation and migration. In particular, this signal transduction pathway is active in response to damage of epidermis, and blockade of ERK activation suppresses keratinocyte migration. Therefore, we investigated the effects of CA extract on stimulating ERK phosphorylation by Western blot analysis. Our data (Figure 6A) revealed that when compared to the untreated group, CA extract rapidly stimulated ERK1/2 phosphorylation (pERK1/2) approximately 3 fold, starting at 5 min after treatment and reaching the maximum activation at 1 h post-treatment. Phosphorylation of ERK1/2 was determined to be approximately 10 fold (Figure 6B). The phosphorylation of ERK1/2 upon CA extract treatment exhibited the unique pattern of a bell-shaped curve where ERK1/2 phosphorylation gradually increased over time and then decreased after 1 h of CA stimulation (Figure 6A,B). Observing ERK1/2 activation led us to the belief that CA may also stimulate the survival signal transduction pathway. Therefore, we detected the activation of Akt by examining phosphorylation of serine 473 of Akt, which is normally responsible for promoting cell survival by inhibiting apoptosis and regulating cell cycle. Data from Western blot analysis showed that CA could rapidly activate Akt phosphorylation, and the phosphorylation pattern was similar to that of pERK1/2. Consistently, PRAS40 which is a downstream substrate of active Akt was also phosphorylated, and the maximal phosphorylation was detected to be between 1 to 3 h post-CA extract treatment (Figure 6A). The rapid activation of ERK1/2 and Akt by CA extract may have occurred as a result of the activation of the upstream molecules of the signaling cascade. Therefore, we detected the activation of epidermal growth factor receptor (EGFR) by targeting tyrosine 1068 (Y1068) phosphorylation and found that CA extract did not activate EGFR receptor, in comparison to the results obtained from HaCaT cells activated with EGF, where EFGR was strongly phosphorylated (Figure 6A).

**Figure 7:**
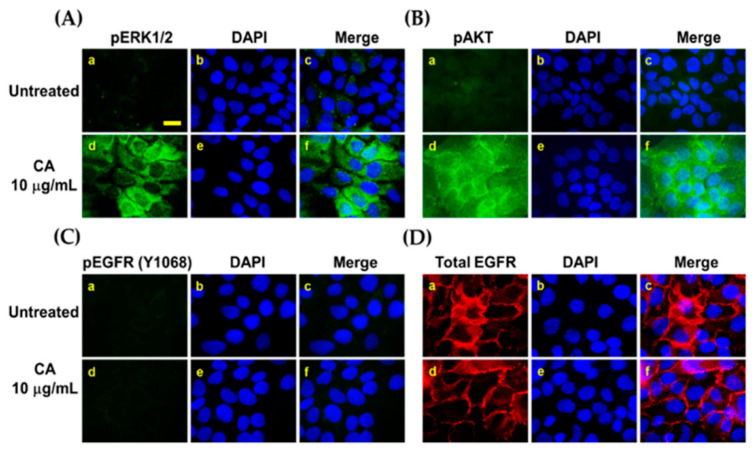
CA extract activated ERK1/2 and Akt phosphorylation in single cells. Immunofluorescence study showing the signal of phosphorylated form of ERK1/2 (green) (**A**) and Akt (green) (**B**) in HaCaT cell treated with 10 µg/mL of CA extract for 15 min. Additionally, phosphorylation status of EGFR at tyrosine 1068 (**C**) and the expression of EGFR protein (red) (**D**) were examined. The nucleus of HaCaT cells were stained with DAPI (blue). Representative pictures were taken by a fluorescent microscope at 100× magnification (scale bar = 200 μm).

Besides Western blot analysis, an immunofluorescence study was done to clearly verify that CA extract activates ERK1/2 and Akt in individual cells. Our studies confirmed that CA extract at 10 µg/mL could strongly induce phosphorylation of ERK1/2 (Figure 7A) and Akt (Figure 7B) in human keratinocytes, but this activation was not seen in the untreated group. Nevertheless, CA extract had no effect on inducing EGFR phosphorylation (Figure 7C) or the receptor’s expression pattern (Figure 7D).

**Figure 8:**
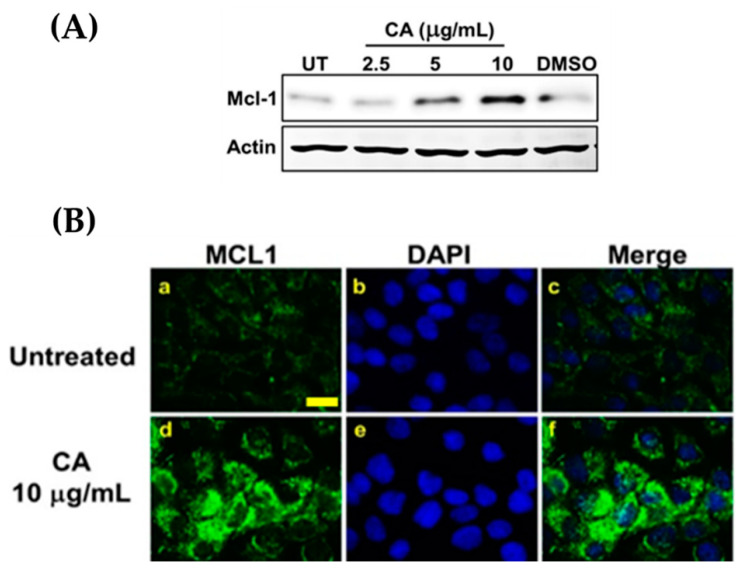
Effects of CA extract on Mcl-1 expression in HaCaT cells detected by Western blot analysis (**A**); and immunofluorescence study (100× magnification, scale bar = 200 μm) (**B**). Data were obtained from three individual experiments.

Additionally, when we detected the expression of Mcl-1, an anti-apoptotic protein in which its expression is regulated by the PI3K/Akt pathways, we found that CA extract induced the expression of this protein in a concentration-dependent manner (Figure 8A). Results from immunofluorescence study verified the findings from Western blot analysis and provided more information on the intracellular location of Mcl-1, which was likely to cluster in the mitochondria where this anti-apoptotic protein normally functions (Figure 8B).

### 3.5. Suppression of ERK1/2 and Akt Activation by Specific Inhibitors Attenuates CA Extract-Induced HaCaT Cell Monolayer Wound Healing

**Figure 9:**
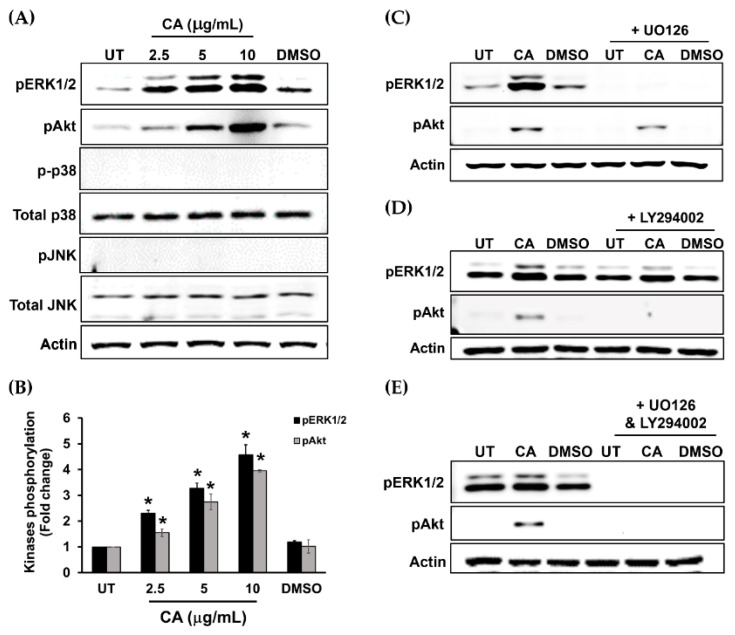
U0126 and LY294002 inhibited CA extract-induced ERK1/2 and Akt phosphorylation. (**A**) Western blot detecting phosphorylated ERK1/2 and Akt in CA-extract-treated HaCaT cells. (**B**) Quantitative analysis of the fold change of ERK1/2 and Akt kinase phosphorylation. (**C**) Western blot analysis for phosphorylated ERK1/2 and Akt in CA-extract-treated HaCaT cells with U0126. (**D**) Western blot detecting phosphorylated ERK1/2 and Akt in CA-extract-treated HaCaT cells with LY294002. (**E**) Western blot detecting phosphorylated ERK1/2 and Akt in CA-extract-treated HaCaT cells with U0126 plus LY294002. Data from three individual experiments present mean ± SD; * *p* < 0.05.

To further confirm the possible mechanism of action of CA extract, we designed additional experiments by using a MEK1 inhibitor (U0126) and a PI3K inhibitor (LY294002) to verify that ERK1/2 and Akt kinases are responsible molecular players in stimulating HaCaT cell proliferation and survival in response to CA extract. Data from Western blot analysis revealed that CA extract could strongly activate ERK1/2 and Akt, but not other MAPKs, including p38 and JNK (Figure 9A,B). As expected, U0126 could specifically inhibit CA extract-induced ERK1/2 phosphorylation (Figure 9C), whereas LY294002 could specifically block Akt phosphorylation (Figure 9D). Moreover, when U0126 and LY294002 were combined, the activation of ERK1/2 and Akt in HaCaT cells stimulated with CA extract was completely inhibited (Figure 9E).

**Figure 10:**
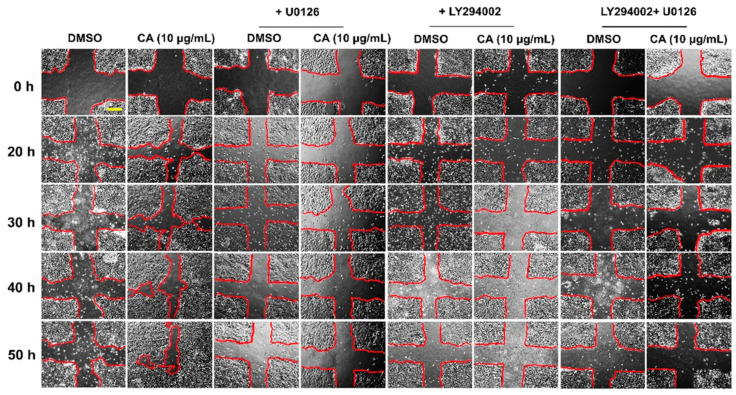
U0126 and LY294002S strongly inhibited the healing-promoting activities of CA extract. Phase-contrast microscopy at 10× magnification (scale bar = 200 μm) was performed to monitor the rate of migration into the wounded site of HaCaT cells treated with CA extract in media containing U0126, LY294002, or the combination of U0126 and LY294002 over the course of 50 h. Regions confined by the red lines indicate the space with no cell occupation. Data are from 3 different experiments.

We next performed a functional test to evaluate whether suppression of growth and survival signaling by U0126 and LY294002 can attenuate healing-enhancing effects of CA extract. Scratch wound healing assay revealed that the effects of CA extract on stimulating HaCaT cell migration were remarkably blocked when U0126 alone, LY294002 alone, or the combination of these two inhibitors were present (Figure 10).

## 4. Discussion

Here we studied *Curcuma amarissima* (CA) ethanolic extract by focusing on its wound healing activities by using HaCaT cell monolayer as a study model, since this cell type is derived from human skin. In particular, we attempted to investigate it to gain concrete evidence for its molecular mechanisms. We first performed a cell viability test by MTT assay to select a range of concentrations that were not toxic to a human epithelial keratinocytes (HaCaT) cell line. Interestingly, results from this experiment where we treated cells with CA extract in FBS-free media clearly showed that CA extract may be able to promote the viability of HaCaT cells. However, this positive effect of CA extract on cell viability was not observed when the treatment system was in complete media where it contained 10% fetal bovine serum (FBS). This phenomenon may be caused by high amount of growth factors in culture media that helps maintain a degree of cell viability so high that it conceals the effects of CA extract. Serum-free condition lacks growth factors, thus allowing its effects on cell viability enhancement to stand out. Data from MTT assay suggest the possibility that CA extract may be able to induce specific cellular events that increase the metabolic activity of the cell, or it may have resulted from increased cell proliferation. As expected, our results from colony-forming assay demonstrated that CA extract could dramatically stimulate the growth of HaCaT colony, suggesting that CA extract may contain potential active constituents that can regulate cell proliferation. Data from crystal violet staining and cell counting clearly confirmed that CA extract promotes cell proliferation to increase a significant number of keratinocytes over time. The possible mechanisms of CA may be similar to those of several growth factors that are critical factors to stimulate the proliferation of human keratinocytes and eventually contribute to efficient healing [35,36]. We then performed a functional test by using a scratch wound model and discovered that CA extract induced a drastic increase in the rate of cell monolayer wound healing. According to our findings, it provides a close correlation between the closure of scratch wound and an increase in HaCaT cell number, suggesting that a contribution to wound healing of CA extract mainly derives from increased cell proliferation.

On the basis that the mitogen-activated protein kinase (MAPK) signaling normally plays crucial roles in cell migration and proliferation regulation [11,12,37,38], we therefore examined whether CA extract contributes to HaCaT cell proliferation through activation of this signal transduction pathway. Interestingly, we disclosed that CA extract stimulated ERK1/2 phosphorylation, and when the phosphorylation of this kinase was inhibited with U0126, the migration rate of HaCaT monolayer was dramatically suppressed. These data strongly suggest that CA extract may possess wound-repairing effects by enhancing cell proliferation and migration, at least in part through ERK1/2 activation. To support our statement about the involvement of ERK1/2 in keratinocyte, many studies previously demonstrated that ERK phosphorylation promoted the migration of this cell type [39,40]. Although ERK1/2 was activated by CA extract, the phosphorylation of p38 kinase and JNK kinase was not affected. These results suggest that not all MAPK signals are involved in CA extract-induced HaCaT cell monolayer healing and indicate that ERK1/2 is the primary signal pathway activated by CA extract. Besides ERK1/2, the phosphatidylinositol 3-kinase (PI3K/Akt) signaling transduction pathway is well characterized to be responsible for migration of many cell types [41,42,43]. Therefore, we determined the effect of CA extract on Akt phosphorylation status in human keratinocytes. Like ERK1/2, we observed a strong increase in phosphorylated Akt level in HaCaT cells incubated with CA extract. Consistent with results from Western blot, an increase in phosphorylation of Akt in individual cells was also confirmed by immunofluorescence study. Additionally, the involvement of this signaling pathway in HaCaT cells induced by CA extract was verified by wound closure assay where a PI3K inhibitor, LY294002, was present. When both U0126 and LY294002 were combined, no monolayer wound healing was evident, indicating that the wound healing activities of CA extract in human keratinocytes are regulated through the activation of the MAPK and PI3K/Akt signal transduction pathways. In addition to the effects on cell proliferation, CA extract has an influence on cell survival by increasing the production of Mcl-1, which is a key anti-apoptotic protein. Mcl-1 production is under the influence of the PI3K/Akt pathway [44]. Moreover, Mcl-1 protein can be upregulated by some cytokines such as IL-6 [45] and growth factors such as EGF, which conveys the cellular signal to control Mcl-1 translation via the MAPK pathway [46,47]. We observed that Mcl-1 was upregulated in individual cells treated with CA extract, and the localization pattern of Mcl-1 suggests that this protein resides in the mitochondria of the cells. Induction of strong expression of Mcl-1 anti-apoptotic protein suggests that CA extract helps increase cell survival of keratinocytes. However, transactivation of epidermal growth factor (EGFR) was not affected by CA extract, indicating that CA extract does not stimulate ERK1/2 and Akt phosphorylation via the EGFR signaling cascade. The activity of CA extract in inducing an increase in cell proliferation and cell survival may be caused by its curcuminoids, including curcumin and demethoxycurcumin. It is evident that curcumin (predominantly found in *Curcuma longa*) stimulates fibroblast proliferation, enhances the formation of granulation tissue, and promotes the contraction and epithelialization of wounds [48,49]. Moreover, the finding that CA extract contains ferulic acid strengthens the possibility to utilize this plant for wound healing since ferulic acid has been documented to be beneficial for skin repair, and this compound has recently been incorporated into the formulation of wound healing [50]. Therefore, our study suggests that CA extract possesses its wound-healing effects, at least in part, through the action of curcuminoids and ferulic acid.

However, our study, using HaCaT cells as a model, has some limitations that require future investigations by other models since HaCaT contains certain genetic alterations that may cause the cell to respond to certain stimuli in a different manner compared to human keratinocyte in actual physiologic conditions. For this reason, human primary keratinocyte should be used to verify the effects of CA extract and its active compound on proliferation and survival. Nevertheless, the use of normal primary human keratinocytes can be limited by the complexities involved in their recovery from donors, cultivation, and limited number of passages. Moreover, primary keratinocytes in vitro have little in common with ordinary naive keratinocytes in vivo under homeostatic conditions. Considering that wounding is a stress that requires several different complex series of communicating processes to respond to various stimuli and to heal the wound [51,52], it needs specific models that can be able to address the promoting effects of a certain agent on wound healing. To overcome these limitations, animal models and clinical trials in humans should be performed in the future to achieve our complete understanding of physiologic and pathologic processes as well as translational efficiency. Altogether, our study revealed that CA extract can potentially trigger and strengthen the molecular healing cascades in human keratinocytes. These events are of great interest for the development of CA as an alternative option for wound healing occurring in some diseases, such as complicated diabetes mellitus, which is less sensitive to treatment by growth factors [53].

## 5. Conclusions

Our present study provides evidence that *Curcuma amarissima* (CA) possesses pharmacological properties in activating human keratinocyte proliferation and survival through its ability to strongly stimulate the phosphorylation of ERK1/2 and Akt kinases. These properties are generally required for promoting wound healing. We demonstrate that this plant contains curcumin, demethoxycurcumin, and ferulic acid, which are potential active compounds reported to be able to promote skin regeneration. Our discovery provides information beneficial for potential uses of CA in regenerative medicine. When mechanisms of action of CA in wound healing is completely defined, and further investigation in animal and human models are done, this plant may be an excellent candidate for the development of a wound-healing agent.

## Data Availability

The data presented in this study are available in this article.

## Supplementary Materials

The following are available online at https://www.mdpi.com/article/10.3390/biology10040289/s1, Figure S1: Chromatographic fingerprint analysis of the ethanolic extract from *Curcuma amarissima* (CA) by high-performance liquid chromatography (HPLC), Figure S2: The HPLC profile of standard ferulic acid and the extract from *Curcuma amarissima*.
