# Supplementary material for: Curcuma amarissima Extract Activates Growth and Survival Signal Transduction Networks to Stimulate Proliferation of Human Keratinocyte
Source: Biology (Basel). 2021 Apr 1;10(4):289. doi: 10.3390/biology10040289 (PMC8067174; doi:10.3390/biology10040289)
Supplement: Supplementary file 1 [file biology-10-00289-s001.pdf]

Supplementary Information

Chromatographic fingerprint analysis of the ethanolic extract from *Curcuma amarissima* (CA) by high-performance liquid chromatography (HPLC)

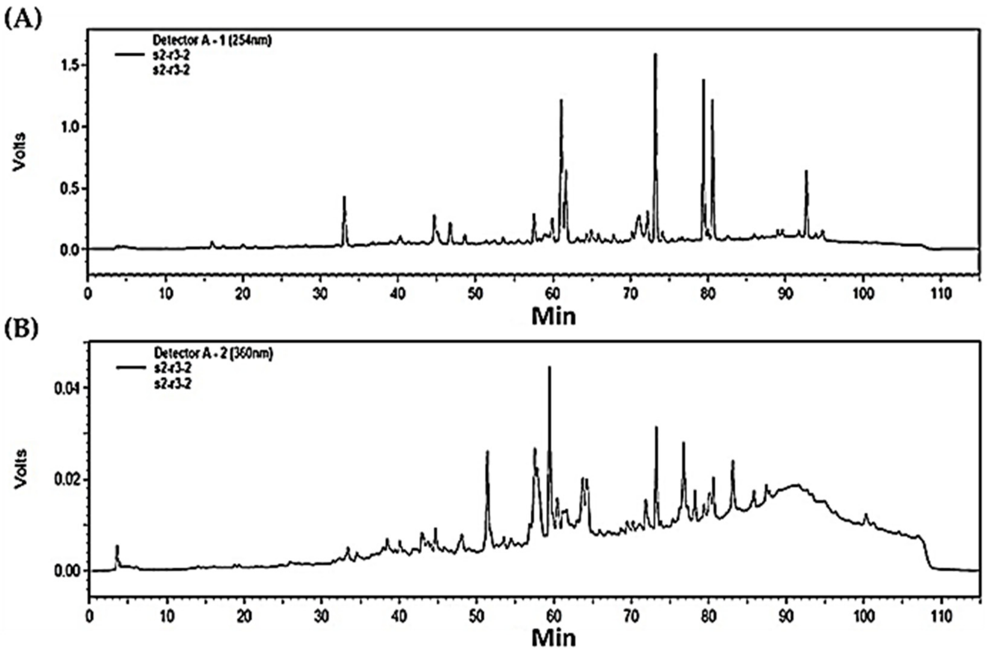

Figure S1. (**A**) HPLC fingerprint of *Curcuma amarissima* (CA) ethanolic extract detected by HPLC/UV at 254 nm; (**B**) HPLC fingerprint of CA ethanolic extract detected by HPLC/UV at 360 nm.

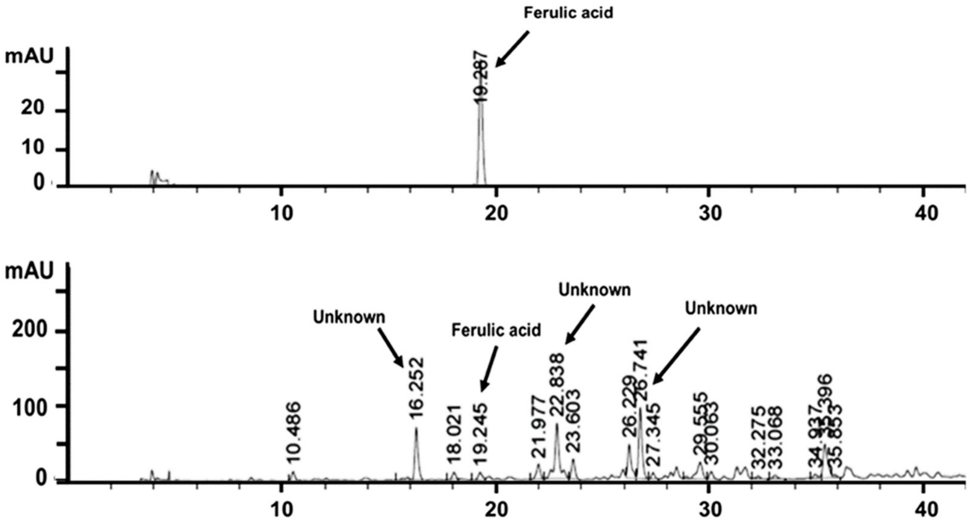

Figure S2. The HPLC profile of standard ferulic acid (20 μg/mL) (upper panel) and the extract from *Curcuma amarissima* (20 mg/mL) (lower panel).
